# Acute hemorrhagic necrotizing pancreatitis in patient with COVID-19: a case report and review of literature

**DOI:** 10.1093/jscr/rjab401

**Published:** 2021-09-22

**Authors:** Sunil Basukala, Kunda Bikram Shah, Bibek Karki, Narayan Thapa, Bikram Basukala, Saurav Karki, Bishnu Deep Pathak

**Affiliations:** Department of Surgery, Nepal Army Institute of Health Science (NAIHS), Kathmandu, Nepal; Department of Surgery, Nepal Army Institute of Health Science (NAIHS), Kathmandu, Nepal; Department of Radio Diagnosis, Nepal Army Institute of Health Science (NAIHS), Kathmandu, Nepal; Department of Surgery, Nepal Army Institute of Health Science (NAIHS), Kathmandu, Nepal; Department of Surgery, Nepal Army Institute of Health Science (NAIHS), Kathmandu, Nepal; Department of Surgery, Nepal Army Institute of Health Science (NAIHS), Kathmandu, Nepal; Department of Surgery, Nepal Army Institute of Health Science (NAIHS), Kathmandu, Nepal

## Abstract

Novel coronavirus disease 2019 (COVID-19) pandemic was originated in Wuhan, China, in December 2019. So far, more than 4 million people worldwide have been infected with the virus. Various manifestations of coronavirus have been reported since the pandemic began. Among them, acute abdomen is one of the manifestations of COVID-19. Some studies have reported acute pancreatitis in several patient due to COVID-19 infection. In this study, we report a rare case in whom SARS-CoV-2 caused acute severe hemorrhagic necrotizing pancreatitis.

## INTRODUCTION

COVID-19, caused by SARS-CoV-2, is an aggressive pulmonary infection that often leads to multi-organ dysfunction. It emerged and was first identified in Wuhan, China in hospitalized patients with pneumonia of unknown origin between December 2019 and January 2020. However, extra-pulmonary manifestations affecting gastrointestinal and hepatobiliary systems, among other systems, have been reported [1]. COVID-19 Although care has primarily focused on the pulmonary system, including viral pneumonia, acute respiratory syndrome (ARDS) and superimposed infections, it is important to recognize the various complications that may arise from patients who are critically ill with this infection. Necrotizing pancreatitis and other manifestations of organ dysfunction should remain on the differential for patients in the ICU [2]. Patients can present with symptoms including anorexia, nausea and/or vomiting, diarrhea and acute abdominal pain [3]. We hereby report a case of COVID-19 resulting in acute hemorrhagic necrotizing pancreatitis presented as acute abdomen in the Emergency department (ED).

## CASE REPORT

A 49-year-old female was brought to the ED for severe abdominal pain that she has experienced over the past 2 days. She described the pain in the epigastric region, which was constant and was radiating to the left the shoulder and was associated with multiple episodes of vomiting without blood. She also revealed that she had fever, cough and shortness of breath for past 1 week for which she self-isolated thinking that she had a coronavirus infection. However, test for coronavirus infection was not performed. Regarding her past medical history, she was diabetic and hypertensive for which she was on medication but had a poor compliance. Upon surgical records, an emergency open appendectomy was done 10 years back. She was non-alcoholic and non-moker. At presentation, she appeared pale, diaphoretic and was in respiratory distress. Physical examination revealed a temperature of 36.3°C, with a heart rate of 130 beats/min, a blood pressure of 130/80 mm of Hg, a respiratory rate of 24 breath/minute and body mass index 29.2 kg/m2. There was no sign of jaundice. On abdominal examination, abdomen was overtly distended with generalized tenderness with no signs of peritonitis. On rectal examination, tone was normal with stool stained in the finger without palpable mass. The remainder of her systemic examination findings was unremarkable. Laboratory findings consistent with acute severe pancreatitis include an elevated amylase, triglyceride and bilirubin. In addition, an increased in white blood cell count with neutrophila, low hemoglobin level and low albumin were noted (Table 1). An arterial blood gas analysis was normal except for mildly raised a lactate level of 2.0.

**Table 1:** Laboratory parameters on admission and during first surgical intervention

s.no	Laboratory test	Normal range	On admission	During laparotomy	Post op
1	WBC count (109 cells/l)	3.5–9.5 10 ^9^ cells/L	14.5 × 10^9^	7.1 × 10^9^	3.9 × 10^9^
2	Neutrophil (%)	50–70%	73	79	83
3	Lymphocyte (%)	20–40%	18	17	08
4	Haemoglobin (g/dl)	11.412–16 g/dl	9.1 g/dl	9.3 g/dl	8.6 g/dl
5	Platelet count (109 cells/l)	125–350 10^9^	180	170	90
6	Haematocrit (%)	36–48%	42	44	35
7	AST (U/l)	5–45 U/l	123	127	149
8	ALT (U/l)	5–40 U/l	97	111	102
9	Albumin (g/l)	3.5–5.5 mg/l	2.6	3.4	3.3
10	Amylase (U/l)	0–140 U/l	1563 U\l	1468 U\l	879 U\l
11	Lipase (U/l)	0–60 U/l	568	491	479
12	Blood sodium level (mEq/l)	135–145 mEq/l	139	137	145
13	Blood potassium level (mEq/l)	3.6–5.2 mEq/l	3.9	3.7	4.7
14	Blood calcium level (mg/dl)	8.5–10.5 mg/dl	6.7	6.9	7.7
15	Triglyceride (mg/dl)	40–150 mg/dl	210	200	219
16	Blood urea nitrogen (mg/dl)	8–20 mg/dl	50	110	120
17	Creatinine (mg/dl)	0.5–1.2 mg/dl	1.2	1.2	3.4

**Figure 1:**
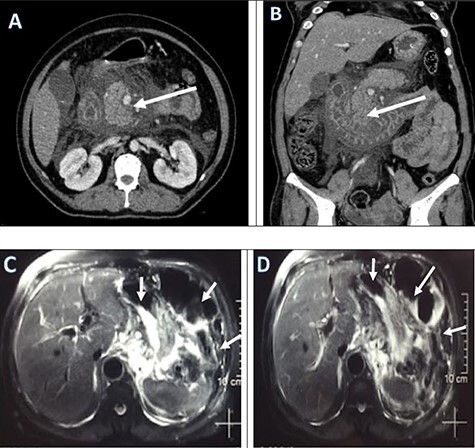
Pre-operative CECT abdomen pelvis (**a, b**) and MRCP (**c, d**) showing pancreatic necrosis and spread of inflammation around the pancreas as shown by red arrows.

Regarding her management at emergency, an aggressive fluid resuscitation with compound sodium lactate 3 litres over half an hour was started. A foley’s catheterization followed by nasogastric tube (NGT) was inserted for gastric decompression. An immediate collection of 900 ml of non-bilious clear fluid was drained. The Rapid Diagnostic Test for COVID 19 infection was sent which showed positive. Then, she was transferred to the COVID-Intensive Care Unit (ICU) for ongoing fluid resuscitation and further management. The PCR test for COVID-19 was sent from ED which showed positive later that day. A broad-spectrum intravenous antibiotics, intravenous analgesics was started. A contrast-enhanced computed tomography (CECT) scan was performed to find out the pathology. Abdominal contrast-enhanced CT showed small indistinct area of hypo-density and hypo-enhancement in the uncinate process with extensive peri-pancreatic inflammation around the pancreas suggestive of acute necrotizing pancreatitis (ANP) (Fig. 1).

**Figure 2:**
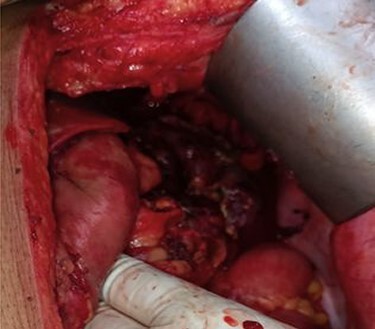
Necrotic pancreatic tissue in the lesser sac undergone necrosectomy.

Thus, the provisional diagnosis of severe Acute Necrotizing Pancreatitis (ANP) was made. Despite all supportive care, her mental status worsened and her clinical status continued to decline. Laboratory parameters showed progressive thrombocytopenia, anemia, hypotension, acute kidney injury (AKI) and metabolic acidosis with respiratory compensation. She had progressive abdominal distension with decreased bowel sounds. The intra-abdominal pressure measured from intra-vesical pressure showed increased (23 mmHg) which leads to the diagnosis of Abdominal Compartment Syndrome (ACS). Thus, a gastrointestinal prokinetic agent and neuromuscular blockade were prescribed. An NGT was inserted to drain the intraluminal content. However, she did not show any improvement with medical treatment; the decompressive laparotomy with a midline incision was planned the following day. Intra-operatively, more than 2 l of ascitic fluid which was brownish to red color was evacuated from the abdominal cavity. There was saponification throughout the mesentery of the transverse colon, and one of the larger areas of saponification was actively draining brownish black pancreatic fluid from the lesser sac. Other findings included severe retroperitoneal edema and an edematous pancreas, with blackish discoloration at the head of pancreas leading the diagnosis of acute hemorrhagic necrotizing pancreatitis (Fig. 2).

**Figure 3:**
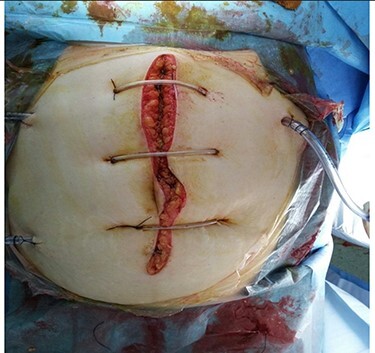
Closure of abdomen with skin retention suture and multiple drains.

Intra-operatively, an extended adhesiolysis was performed with opening of gastrocolic ligament and followed by necrotic debridement, and drainage placement. Two radiopaque 28 F abdominal drains were placed in the lesser sac and at the base of the mesentery of the transverse colon and one at the pelvis. The abdomen was left open and was skin opposed with retention sutures (Fig. 3). The patient underwent post-operative resuscitation in the ICU.

During the post-operative period, the patient became progressively difficult to ventilate with very poor pulmonary compliance. She was transfused four pints of whole blood during her stay in ICU. Tidal volume was less than 250 ml on a driving pressure of 15 and a PEEP of 15. PaO_2_ remained in the 65 s despite FiO^2^ of 1.0. She was unresponsive to fluid challenge and required an infusion of 45 mcg/min of noradrenaline and 2 mcg/min of vasopressin in order to maintain an adequate mean arterial pressure. Gradually patient developed AKI, being oliguric (<10 ml/h) with a potassium of 6.5 mmol/l. Her blood parameters showed sepsis with WBC count of 3200 × 10^9^. Despite correction of ACS and active intensive therapy, the patient’s condition did not improve and she died 2 days after laparotomy as a result of multi-organ failure.

## DISCUSSSION

The novel severe ARDS coronavirus 2 (SARSCoV2) has led to an unprecedented global health crisis. The severity of the disease ranges from subclinical infections to severe illness requiring critical care measures, and fatality rate is reported to reach as high as 15% in some countries [4, 5].

This report illustrates a patient requiring critical care very soon after presentation with acute abdomen, with an ultimate finding of acute hemorrhagic necrotizing pancreatitis. COVID-19 disease also has its impact on the gastrointestinal tract in less than 10% of cases which include nausea/vomiting (7.8%), diarrhoea (7.7%), abdominal pain (2.7%) and liver enzyme abnormalities (15%). Initially, there appeared to be no link between the SARS-CoV-2 virus and the pancreas. Since then, a few cases of COVID-19 disease presenting as acute pancreatitis have been reported. Several mechanisms of pancreatic injury have been described, such as direct cytopathic effect of the virus and dysregulated immune response induced by SARS-CoV-2 that targets the pancreas in addition to the lungs [6, 7]. Liu *et al.* [8] also showed 17% incidence of the pancreatic injury in 67 severe COVID-19 cases although the injury was evident on CT scan in only 7.46% cases [8]. Anand *et al.* [9] reported a case of COVID-19 with acute pancreatitis who initially presented with fever, cough, a sore throat and myalgia [9]. The Acute Pancreatitis (AP) was diagnosed on CT scan that showed diffusely edematous pancreatitis. Very few studies have mentioned necrotizing AP even though some were classified as severe pancreatitis, and none required any intervention for pancreatitis-related local complications. A study done by Baron TH *et al.* [10] has stated the hemorrhagic complication among patient with ANP in patient infected with COVID-19. This study also stated that the development of acute hemorrhagic necrotizing pancreatitis in patients COVID-19 carries the risk of erosive hemorrhage from pancreas due to coagulopathy. Precise monitoring of coagulation and fibrinolytic activity is highly indicated among these patients to reduce morbidity and mortality of the patient [10]. Mortality of acute hemorrhagic necrotizing pancreatitis is up to 20–30% or even higher up to 70–93% in the presence of infected necrosis [11]. Septic shock, sepsis, acute renal failure and coagulopathies are poor prognostic factors. The Ranson’s score and Balthazar’s criteria are reliable in predicting prognosis although many other predictors of severity [12] have been tested to make progress in early detection of complications. On average, 50% of the severe form of pancreatitis with infected necrosis or hemorrhage die post-operatively [13] and some literature reports no survivors at all, in those treated without operation [11]. This suggests the usefulness of early surgical treatment once infected pancreatic necrosis is suspected. However, the advisability of operation and the prognostic implications derived from the morphologic categorization of acute hemorrhagic necrotizing pancreatitis are applicable only retrospectively after the operation or autopsy.

Current studies [14] show no superiority of prognostic assessment using CT scans as opposed to clinical and biochemical evaluation in the initial phase of the disease, thus thorough clinical assessment is the single most important tool in developing countries, where CT scans and MRI are not feasible. Whenever in doubt, it is important to remember that minimally invasive approaches and/or laparotomy, with continuous post-operative peritoneal lavage, are the main surgical interventions for infected hemorrhagic necrotic pancreatitis, with better outcome [14, 15].

## CONCLUSION

COVID-19 is an aggressive pulmonary infection that often leads to multi-organ dysfunction. Although much attention is given to treating superimposed infection, ventilatory and pulmonary support, it is important to monitor patients closely for additional, less common sequelae of the disease. Hence, acute hemorrhagic necrotizing pancreatitis should be suspected among patient with ANP in patient infected with COVID-19 who shows no improvement with conservative treatment.
